# Penetration of echinocandins into wound secretion of critically ill patients

**DOI:** 10.1007/s15010-021-01604-x

**Published:** 2021-04-20

**Authors:** Tiziana Gasperetti, René Welte, Herbert Oberacher, Jana Marx, Ingo Lorenz, Peter Schellongowski, Thomas Staudinger, Karin Burgmann, Philipp Eller, Tobias Santner, Andrea Griesmacher, Hartwig Pfisterer, Stephan Eschertzhuber, Maria Aigner, Michael Joannidis, Romuald Bellmann

**Affiliations:** 1 Clinical Pharmacokinetics Unit, Division of Intensive Care and Emergency Medicine, Department of Internal Medicine I, Medical University of Innsbruck, Anichstrasse 35, 6020 Innsbruck, Austria; 2 Institute of Legal Medicine and Core Facility Metabolomics, Medical University of Innsbruck, Innsbruck, Austria; 3 General and Surgical ICU, University Hospital for Anaesthesia and Intensive Care, Medical University of Innsbruck, Innsbruck, Austria; 4 Intensive Care Unit, Department of Internal Medicine I, Medical University of Vienna, Vienna, Austria; 5 Intensive Care Unit, Department of Internal Medicine, University Hospital of Graz, Graz, Austria; 6 Central Institute for Medical and Chemical Laboratory Diagnostics, Innsbruck General Hospital, Innsbruck, Austria; 7 Transplant ICU, University Hospital for Anaesthesia and Intensive Care, Innsbruck General Hospital and Innsbruck Medical University Innsbruck, Innsbruck, Austria; 8 Institute of Hygiene and Medical Microbiology, Medical University of Innsbruck, Innsbruck, Austria; 9 Division of Intensive Care and Emergency Medicine, Department of Internal Medicine I, Medical University of Innsbruck, Innsbruck, Austria; 10 Present Address: Department of Anaesthesia and Critical Care, District Hospital of Hall in Tyrol, Hall in Tyrol, Austria; 11 Present Address: INNPATH GmbH, Tirol Kliniken, Innsbruck, Austria

**Keywords:** Echinocandin antifungals, Target-site pharmacokinetics, Wound infection, Invasive candidiasis, Vacuum assisted closure therapy

## Abstract

**Purpose:**

Wound infections caused by *Candida* are life-threatening and difficult to treat. Echinocandins are highly effective against *Candida* species and recommended for treatment of invasive candidiasis. As penetration of echinocandins into wounds is largely unknown, we measured the concentrations of the echinocandins anidulafungin (AFG), micafungin (MFG), and caspofungin (CAS) in wound secretion (WS) and in plasma of critically ill patients.

**Methods:**

We included critically ill adults with an indwelling wound drainage or undergoing vacuum-assisted closure therapy, who were treated with an echinocandin for suspected or proven invasive fungal infection. Concentrations were measured by liquid chromatography with UV (AFG and MFG) or tandem mass spectrometry detection (CAS).

**Results:**

Twenty-one patients were enrolled. From eight patients, serial WS samples and simultaneous plasma samples were obtained within a dosage interval. AFG concentrations in WS amounted to < 0.025–2.25 mg/L, MFG concentrations were 0.025–2.53 mg/L, and CAS achieved concentrations of 0.18–4.04 mg/L. Concentrations in WS were significantly lower than the simultaneous plasma concentrations and below the MIC values of some relevant pathogens.

**Conclusion:**

Echinocandin penetration into WS displays a high inter-individual variability. In WS of some of the patients, concentrations may be sub-therapeutic. However, the relevance of sub-therapeutic concentrations is unknown as no correlation has been established between concentration data and clinical outcome. Nevertheless, in the absence of clinical outcome studies, our data do not support the use of echinocandins at standard doses for the treatment of fungal wound infections, but underline the pivotal role of surgical debridement.

**Supplementary Information:**

The online version contains supplementary material available at 10.1007/s15010-021-01604-x.

## Introduction

Wound infections by *Candida* occur mainly after surgery, severe burns or traumatic injuries [1–3]. The outcome of this devastating and life-threatening condition largely depends on surgical debridement and on appropriate antifungal treatment [4]. However, the optimal antifungal drug regimen for *Candida* wound infections remains to be established [5]. Echinocandins are cyclic hexapeptides, which are highly effective against most of the pathogenic *Candida* species. Based on their efficacy against candidemia, echinocandins are recommended for the treatment of invasive candidiasis by current guidelines [6]. However, there is only one report on wound penetration of an echinocandin [7]. Therefore, we measured the concentrations of the commercially available echinocandins anidulafungin (AFG), micafungin (MFG), and caspofungin (CAS) in wound secretion (WS) and in simultaneously drawn plasma samples of critically ill patients treated with AFG, MFG, or CAS for suspected or proven invasive fungal infection.

## Study population and methods

### Study design and patient enrolment

This was an open-label, pharmacokinetic multi-centre study. The protocol was approved by the local ethics committees (EudraCT no. 2013-005065-38), and the study was carried out in accordance with the Declaration of Helsinki and with Austrian law. Written informed consent was obtained from competent patients, *post-hoc* consent from patients who were incompetent at the time of enrolment.

Consecutive critically ill adults were eligible if they fulfilled the following inclusion criteria: (1) ongoing echinocandin treatment with either AFG, MFG, or CAS for suspected or proven invasive candidiasis, and (2) indwelling wound drainage at any anatomical site or ongoing vacuum-assisted closure (V.A.C.) therapy.

### Echinocandin treatment

AFG (Ecalta®; Pfizer, Sandwich, Kent, UK), MFG (Mycamine®; Astellas, Leiderdorp, NL), and CAS (Cancidas®; Merck Sharp and Dohme, Hoddesdon, Hertfordshire, UK), respectively, were administered at the discretion of the treating physician. As recommended by the manufacturer, the daily maintenance dose of AFG amounted to 100 mg after a 200-mg loading dose. MFG was given at a daily dose of 100 mg. The standard maintenance dose of CAS is 50 mg daily after a single-loading dose of 70 mg. Patients with a body weight above 80 kg should receive 70 mg daily during the entire treatment.

### Sampling and echinocandin quantification

Samples were taken after the first echinocandin dose or after multiple doses in accordance with clinical requirements and the availability of WS. When WS was continuously drained into collection bags, a highly variable delivery of WS had to be considered. Whenever sufficient amounts of WS were yielded within a dosage interval, the bags were changed before the echinocandin infusion, as well as at 1, 4, 8, 12, 18, and 24 h after the start of infusion, and kept for analysis. When delivery of WS was insufficient for serial sampling (< 0.5 mL), the collection bag was changed only once or twice, and taken for echinocandin quantification. In the case of incomplete serial sampling on the first study day due to poor WS delivery, additional samples were taken the following day, if available. From patients with two or three indwelling wound drainages, WS was sampled simultaneously from these drains, if available. In patients undergoing V.A.C. therapy, the change of the V.A.C. container was scheduled according to the clinical requirements and kept for echinocandin quantification. Simultaneously with the change of the collection bags or V.A.C. containers, 2 mL of blood was drawn from the arterial line using heparinized vials (Sarstedt, Nümbrecht, Germany). Whole blood was centrifuged at 350 × *g* for 10 min to obtain plasma. WS and plasma were stored at − 80 °C until analysis. As detailed in Online Resource 1, AFG and MFG were quantified by high-performance liquid chromatography with UV detection [8]; whereas, CAS concentrations were measured by means of liquid chromatography-tandem mass spectrometry (LC–MS/MS).

### Data analysis

Echinocandin pharmacokinetics were calculated by a non-compartmental model using Kinetica 2000® (InnaPhase Corporation, Champs-sur-Marne, France). The area under the concentration–time curve from the start of the echinocandin infusion to the last sampling (AUC_0-n_) was computed using the log-linear method when the concentration in a trapezoid decreased, or with the trapezoidal method if the concentration increased. When serial WS samples had been obtained within the dosage interval, the penetration ratio (PR) was defined as the ratio between the AUC_0-n_ over the same sampling period in WS and in plasma (AUC_0-n__ws_/AUC_0-n__plasma_). For single samples, the PR was the ratio between the WS concentration and the plasma concentration (*C*_ws_/*C*_plasma_). The significance of the differences between WS and plasma concentrations of AFG, MFG, or CAS was calculated with the Wilcoxon matched-pairs test. The significance of the differences between the concentrations of AFG, MFG, and CAS and between the PRs of the three echinocandins was assessed with the Mann–Whitney *U* test and Bonferroni correction. PRs calculated from AUC_0-n__ws_/AUC_0-n__plasma_ and *C*_ws_/*C*_plasma_ were considered in equal measure. The IBM SPSS® Statistics software version 26.0 (Armonk, NY, USA) was used for the statistical calculations.

## Results

### Study population

Twenty-one patients were enrolled in this study. Eleven were females. The characteristics of the study patients and the detail of sampling are summarized in Online Resource 2. A total of 70 sample pairs (WS and plasma) were analysed. Serial sample pairs, allowing for calculation of echinocandin pharmacokinetics in WS and in plasma, were obtained from eight patients. Three of these patients were on AFG, three on MFG, and two on CAS. From seventeen patients, single sample pairs were collected at different times from echinocandin infusion (Online Resource 2, Tables 1, 2 and 3). *Candida* species were isolated from WS of nine study patients (Online Resource 2). Minimal inhibitory concentrations (MICs) of six isolates were determined by E-test® and amounted to 0.002–0.38 mg/L. Four patients presented candidemia. Ten patients were discharged from hospital within 4 months after start of echinocandin therapy, while eleven patients died in ICU or in hospital (Online Resource 2).

**Table 1:** Anidulafungin in serial samples and single samples of wound secretion and plasma

Serial sampling										
Patient No	AFG dose (mg/day)	Day of AFG treatment	Sampling period (h)	Wound secretion			Plasma			PR
				*C*_max_ (mg/L)	*T*_max_ (h)	AUC_0-n_ (mg × h/L)	*C*_max_ (mg/L)	*T*_max_ (h)	AUC_0-n_ (mg × h/L)	
1	MD, 100 (LD, 200)	3	24	0.33	8	6.82	4.04	1	60.72	0.11
2^a^	MD, 100 (LD, 200)	1	12	2.25	8	14.91	9.46	1	53.76	0.28
3^b^	MD, 100 (LD, 200)	11	24	1.74	0	25.47	6.53	1.5	116.19	0.22
Median		3	24	1.74	8	14.91	6.53	1	60.72	0.22
Range		1–11	12–24	0.33–2.25	0–8	6.82–25.47	4.04–9.46	1–1.5	53.76–116.19	0.11–0.28

Single sampling						
Patient No	AFG dose (mg/day)	Day of AFG treatment	Time from infusion (h)	Wound secretion	Plasma	PR
				AFG concentration (mg/L)	AFG concentration (mg/L)	
2^a^	MD, 100 (LD, 200)	2	2	2.21	4.19	0.53
			6.5	1.30	3.51	0.37
3^b^	MD, 100 (LD, 200)	10	21.5	1.94	4.04	0.48
4^c^	MD, 100 (LD, 200)	16	3	0.81	2.77	0.29
5	MD, 100 (LD, 200)	17	4	1.71	3.19	0.54
6	MD, 100 (LD, 200)	3	15.5	0.31	1.55	0.20
7^c,d^	MD, 100 (LD, 200)	2	27	< 0.025	0.98	< 0.026
Median		6.5	6.5	1.30	3.19	0.37
Range		2–17	2–27	< 0.025–2.21	0.98–4.19	< 0.026–0.54

*AFG* anidulafungin, *MD* maintenance dose, *LD* loading dose, *Sampling period* time from the first to the last sampling, *Time from infusion* time from the start of the echinocandin infusion to sampling of wound secretion and plasma, *C*_*max*_ echinocandin peak concentration, *T*_*max*_ time to *C*_max_, *AUC*_*0-n*_ area under the concentration–time curve from the start of the echinocandin infusion to the last sampling, *PR* penetration ratio

^a^For patient 2, serial sampling on the first study day was incomplete, therefore, two additional sample pairs were taken one day later

^b^For patient 3, only one single sample pair could be obtained on day 10 of AFG therapy, but serial sampling was possible the following day

^c^Wound secretion had been collected over 17 h (patient 4) or 80 h (patient 7) in a vacuum-assisted closure (V.A.C.) canister containing an absorption gel

^d^The AFG concentration in wound secretion was < 0.025 mg/L, probably because the V.A.C. canister had been installed 27 h before the initiation of echinocandin therapy

**Table 2:** Micafungin in serial samples and single samples of wound secretion and plasma

Serial sampling										
Patient No	MFG dose (mg/day)	Day of MFG treatment	Sampling period (h)	Wound secretion			Plasma			PR
				*C*_max_ (mg/L)	*T*_max_ (h)	AUC_0-n_ (mg × h/L)	*C*_max_ (mg/L)	*T*_max_ (h)	AUC_0-n_ (mg × h/L)	
8^a^	100	19	24	1.69	12	24.46	7.20	1	72.30	0.34
				2.53	12	52.06	7.20	1	72.30	0.72
9^b^	100	6	24	0.97	12	18.41	7.43	1	89.50	0.21
10^b^	100	3	24	0.09	8	1.41	4.27	1	40.36	0.03
Median		6	24	1.33	12	21.43	7.20	1	72.30	0.27
Range		3–19	24–24	0.09–2.53	8–12	1.41–52.06	4.27–7.43	1–1	40.36–89.50	0.03–0.72

Single sampling						
Patient No	MFG dose (mg/day)	Day of MFG treatment	Time from infusion (h)	Wound secretion	Plasma	PR
				MFG concentration (mg/L)	MFG concentration (mg/L)	
9^b^	100	6	24	0.65	2.16	0.30
10^b^	100	3	24	0.025	0.78	0.032
11^c^	100	5	21	1.14	0.34	3.36
12^d^	100	2	24	0.41	0.80	0.51
				0.41	0.80	0.51
				0.41	0.80	0.51
13	100	5	24	0.48	4.57	0.11
Median		3	24	0.41	0.80	0.30
Range		2–6	21–24	0.025–1.14	0.34–4.57	0.03–3.36

*MFG* micafungin, *MD* maintenance dose, *LD* loading dose, *Sampling period* time from the first to the last sampling; *Time from infusion* time from the start of the echinocandin infusion to sampling of wound secretion and plasma, *C*_*max*_ echinocandin peak concentration, *T*_*max*_ time to *C*_max_, *AUC*_*0-n*_ area under the concentration–time curve from the start of the echinocandin infusion to the last sampling, *PR* penetration ratio

^a^For patient 8, two wound secretion drainages had been inserted at two abdominal sites

^b^The wound secretion samples of patient 9 and 10 were taken from two different drainages on the same study day. Serial samples were collected from one of the two drainages, while only one single sample was obtained from the other one

^c^For patient 11, wound secretion had been collected in a vacuum-assisted closure (V.A.C.) canister containing an absorption gel and held in place for 43 h

^d^For patient 12, three wound secretion samples were collected at the same sampling time from three different mediastinal drains

**Table 3:** Caspofungin in serial samples and single samples of wound secretion and plasma

Serial sampling										
Patient No	CAS dose (mg/day)	Day of CAS treatment	Sampling period (h)	Wound secretion			Plasma			PR
				*C*_max_ (mg/L)	*T*_max_ (h)	AUC_0-n_ (mg × h/L)	*C*_max_ (mg/L)	*T*_max_ (h)	AUC_0-n_ (mg × h/L)	
14	MD, 50 (LD, 70)	3	18	3.24	12	53.28	6.74	1	70.46	0.76
15	70	7	24	2.99	18	56.90	23.60	1	277.97	0.20
Median		5	21	3.12	15	55.09	15.17	1	174.21	0.48
Range		3–7	18–24	2.99–3.24	12–18	53.28–56.90	6.74–23.60	1–1	70.46–277.97	0.20–0.76

Single sampling						
Patient No	CAS dose (mg/day)	Day of CAS treatment	Time from infusion (h)	Wound secretion	Plasma	PR
				CAS concentration (mg/L)	CAS concentration (mg/L)	
16	MD, 50 (LD, 70)	7	15	0.46	3.36	0.14
17	MD, 50 (LD, 70)	2	24	4.04	4.94	0.82
18	MD, 50 (LD, 70)	2	6	0.39	8.11	0.05
19^a^	MD, 50 (LD, 70)	15	2.5	0.69	17.68	0.04
			22	0.53	5.77	0.09
20^b^	MD, 50 (LD, 70)	1	11	0.18	1.91	0.09
		2	2	1.77	9.93	0.18
21	70	9	18	1.30	3.90	0.33
Median		4.5	13	0.61	6.94	0.12
Range		1–15	2–24	0.18–4.04	1.91–17.68	0.04–0.82

*CAS* caspofungin, *MD* maintenance dose, *LD* loading dose, *Sampling period* time from the first to the last sampling, *Time from infusion* time from the start of the echinocandin infusion to sampling of wound secretion and plasma, *C*_*max*_ echinocandin peak concentration, *T*_*max*_ time to *C*_max_, *AUC*_*0-n*_, area under the concentration–time curve from the start of the echinocandin infusion to the last sampling, *PR* penetration ratio

^a^For patient 19, two wound secretion samples were collected on the 15th day of CAS treatment from the same sternal site

^b^For patient 20, two wound secretion samples were collected at different days of CAS therapy from the same intra-abdominal drain

### Echinocandin concentrations and pharmacokinetics

In WS, echinocandin concentrations were lower than in plasma (*P* value < 0.0001). The AFG concentrations in WS samples ranged from < 0.025–2.25 mg/L, and the MFG concentrations were 0.025–2.53 mg/L. In the corresponding plasma samples, the AFG concentrations were 0.98–9.46 mg/L, and the MFG concentrations amounted to 0.34–7.43 mg/L. CAS achieved higher concentrations with 0.18–4.04 mg/L in WS and 1.84–23.60 mg/L in plasma (*P* value < 0.002 and < 0.05, respectively). There was no significant difference in PRs between the three echinocandins (*P* value > 0.05, Tables 1, 2 and 3).

Serial sampling revealed a slower rise and decline of echinocandin concentrations in WS than in plasma. The area under the concentration–time curve over 24 h (AUC_0-24_) for AFG in WS was 6.82 and 25.47 mg × h/L on day 3 and 11, respectively, and amounted to 1.41 and 52.06 mg × h/L for MFG on day 3 and 19, respectively. For CAS, an AUC_0-18_ of 53.28 and an AUC_0-24_ of 56.90 mg × h/L were determined (treatment day 3 and 7, respectively). In plasma, the respective AUC values amounted to 53.76–116.19 mg × h/L for AFG, 40.36–89.50 mg × h/L for MFG, and 70.46–277.97 mg × h/L for CAS (Tables 1, 2 and 3).

The single sample pairs, which were taken on various treatment days and at different time from infusion, yielded highly variable echinocandin concentrations and PRs. The median PR was 0.37 for AFG, 0.30 for MFG, and 0.12 for CAS (differences not significant, see Tables1, 2 and 3).

## Discussion

Yamada et al. reported a MFG concentration of 4.42 (3.90–4.93) mg/L with a PR of 0.46 (0.40–0.51) [median (range)] in WS of a critically ill patient, two to four hours after infusion of a 150-mg dose at steady state [7]. So far, this has been the only report on penetration of an echinocandin into WS. A MFG concentration of 0.38 mg/L was measured in pancreatic pseudocyst fluid on the seventh day of therapy with 100 mg of MFG daily [9]. In burn eschar, MFG reached median concentrations of 0.5–4.0 mg/L [10–13]. A necrotizing fasciitis caused by *C. albicans* following thyroidectomy healed under AFG therapy combined with surgical debridement [1]. Azoles and amphotericin B were also applied for treatment of fungal wound infections [14]. In WS of two patients treated with liposomal amphotericin B (5 mg/kg daily) for 4 and 15 days, respectively, amphotericin B concentrations were between 0.2 and 3.0 mg/L. Voriconazole achieved concentrations of 0.6–2.7 mg/L in WS of one of the patients [15].

MICs of echinocandins were determined in *Candida* isolates from wounds of six of our study patients. All the MICs were below the echinocandin concentrations measured in WS of these patients. *In-vitro* MIC values of echinocandins range from ≤ 0.008 to 1.0 mg/L for *C. albicans* and *C. glabrata*, from 0.03 to 0.25 mg/L for *C. krusei*, and from ≤ 0.5 to 2.0 mg/L for *C. lusitaniae* [16]. The pharmacokinetic-pharmacodynamic target parameter that best correlates with the efficacy of echinocandin treatment is the ratio between the AUC_0-24_ and the MIC of the pathogen (AUC_0-24_/MIC) [17, 18]. Andes et al. reported a fungistatic effect on *Candida* species for AUC_0-24_/MIC ratios exceeding 2,782 for AFG, 5,299 for MFG, and 748 for CAS [17]. We calculated this ratio when serial samples over 24 h had been obtained and *Candida* had been cultivated from a wound. The AUC_0-24_/MIC ratio achieved in patient 3, who had been treated with AFG, and in patient 15 treated with CAS, amounted to 1,592, and 14,225, respectively, suggesting an adequate local exposure for patient 15, but not for patient 3.

Some limitations of our study must be considered. WS was collected from different anatomical sites via conventional drainage or during V.A.C. therapy, resulting in considerable heterogeneity of WS. The high viscosity of some of the WS samples hampered the measurement. Differences in WS composition, particularly in protein and lipid content, might have affected the distribution of echinocandins [19]. The plasma protein binding of CAS amounts to 96% which is lower than that of AFG (99%) and MFG (99.9%) [19]. In the present study, we measured the total drug concentration only, without discrimination between the protein-bound and free echinocandin fraction. The larger free fraction of CAS might favour target site penetration. Accordingly, CAS has achieved higher WS concentration than AFG and MFG. But the PR of CAS was not significantly different from the PRs of AFG and MFG, probably, because also the plasma concentrations of CAS exceeded AFG and MFG plasma levels. In WS, however, the extent of protein binding of echinocandins is unknown, but it might largely affect their antifungal activity. Our study population comprised of critically ill patients presenting with quite different underlying diseases, comorbidities, and constitution. This might have contributed to the high inter-individual variability of echinocandin WS concentrations [20–23]. This variability impedes the interpretation of our findings but largely reflects the situation in critical care medicine. In critically ill patients, highly variable pharmacokinetics of AFG, MFG, and CAS was reported even for serum pharmacokinetics [24]. Furthermore, samples were taken after different treatment duration, and six of our twenty-one study patients (patient 1, 2, 6, 7, 12, and 20) had not yet reached steady state. From thirteen patients, we obtained only single sample pairs at different times from echinocandin infusion. Thus, the interval between administration and sampling affected PR because of hysteresis [25]. V.A.C. canisters were in place for up to 80 h (patient 7, day 2 of AFG therapy). Echinocandin concentrations measured in these samples might, therefore, reflect average WS concentration that was influenced by significant dilution. Chemical degradation of echinocandins cannot be ruled out in WS obtained from V.A.C. canisters. The relevance of in-vitro MIC values for WS is unknown. Thus, WS concentrations exceeding in-vitro MICs do not prove their antifungal efficacy at target-site. Only four of our study patients presented candidemia. *Candida* was isolated from WS of nine study patients representing probably colonization. *Candida* was cultivated from both blood and WS of two patients. Our study addressed target-site penetration and pharmacokinetics of echinocandins and was, therefore, not designed and not powered for the assessment of clinical outcome.

Controlled clinical trials on medical treatment of fungal wound infections have not yet been published. The few available data on WS penetration of antifungal drugs as well as the results of the present study suggest a highly variable but limited accessibility of WS. Reliable efficacy of echinocandins applied at standard doses against fungal wound infections can be anticipated only when the causative pathogen is highly susceptible. Thus, timely and thorough surgical debridement has probably a pivotal role in this condition. In addition, high-dose echinocandin treatment, e.g. caspofungin 150 mg daily, could be considered under close monitoring for toxicity [26]. This approach, however, will require evaluation in clinical outcome studies.

## Conclusions

Echinocandin penetration into WS displays a high inter-individual variability. In WS of some of the patients, concentrations may be sub-therapeutic. However, the relevance of sub-therapeutic concentrations is unknown as no correlation has been established between concentration data and clinical outcome. Nevertheless, in the absence of clinical outcome studies, our data do not support the use of echinocandins at standard doses for the treatment of fungal wound infections but underline the pivotal role of surgical debridement.

## Supplementary Information

Below is the link to the electronic supplementary material.

Supplementary file1 (PDF 40 KB)

Supplementary file2 (PDF 129 KB)

## Data Availability

Echinocandin pharmacokinetics were calculated using Kinetica 2000® (InnaPhase Corporation, Champs-sur-Marne, France). Statistical analysis was conducted using IBM SPSS® Statistics software version 26.0 (Armonk, NY, USA).

## Declarations

### Availability of data and material

The datasets used and analysed during the current study are available from the corresponding author on request.
