# Supplementary material for: Penetration of echinocandins into wound secretion of critically ill patients
Source: Infection. 2021 Apr 20;49(4):747–55. doi: 10.1007/s15010-021-01604-x (PMC8316195; doi:10.1007/s15010-021-01604-x)
Supplement: Supplementary file 1 — Supplementary file1 (PDF 40 KB) [file 15010_2021_1604_MOESM1_ESM.pdf]

**ELECTRONIC SUPPLEMENTARY MATERIAL 2**

# Penetration of Echinocandins into Wound Secretion of Critically Ill Patients

**Journal name: Infection**

Tiziana Gasperetti, René Welte, Herbert Oberacher, Jana Marx, Ingo Lorenz, Peter Schellongowski, Thomas Staudinger, Karin Burgmann, Philipp Eller, Tobias Santner, Andrea Griesmacher, Hartwig Pfisterer, Stephan Eschertzhuber, Maria Aigner, Michael Joannidis, Romuald Bellmann

Corresponding author: Romuald Bellmann, MD, associate professor,

Clinical Pharmacokinetics Unit, Division of Intensive Care and Emergency Medicine,

Department of Internal Medicine I, Medical University of Innsbruck,

Anichstrasse 35, 6020 Innsbruck, Austria, Europe,

romuald.bellmann@i-med.ac.at, Phone: 0043 512 504 81389, Fax: 0043 512 504 67 81389

**ONLINE RESOURCE 2** Study population and echinocandin treatment

| **Patients on anidulafungin** | | | | | | |
|---|---|---|---|---|---|---|
| **Patient No.** | **Body weight (kg)** | **Cum. dose (mg)** | **WS and plasma samples obtained** | **Localization and type of drainage** | **Main diagnosis** | **Clinical outcome** |
| 1 | 75 | 400 | Serial samples (5) | Thigh (drain) | Gangrenous phlegmon (shank), candidemia (*C. albicans* and *C. glabrata*) | Deceased in ICU |
| 2 | 67 | 200 | Serial samples (5), 2 single samples | Retroperitoneal (drain) | Duodenal adenocarcinoma, st. p. pancreatectomy and splenectomy, suspected wound infection (*C. albicans* from WS drain, MIC = 0.002 mg/L) | Discharged from hospital |
| 3 | 46 | 1,100 | 1 single sample, serial samples (7) | Surgical wound at porta hepatis (drain) | Candidemia (*C. krusei*), suspected wound infection (*C. krusei* from wound drain tip, MIC = 0.016 mg/L), st. p. LTX, TBC | Deceased in ICU |
| 4 | 60 | 1,700 | 1 single sample | Subcutaneous abdominal (V.A.C.) | Candidemia (*C. krusei*), suspected abdominal wound infection (*C. krusei* from | Deceased in ICU |

| | | | | | | |
|---|---|---|---|---|---|---|
| | | | | | V.A.C., MIC = 0.016 mg/L), ovarian cancer, peritoneal carcinosis | |
| 5 | 70 | 1,800 | 1 single sample | Intraabdominal (V.A.C.) | Peritonitis, partial anastomotic dehiscence, gastric cancer relapse, suspected wound infection | Deceased in ICU |
| 6 | 95 | 400 | 1 single sample | Thigh (V.A.C.) | Septic arthritis, thigh abscess, SLE | Deceased in hospital |
| 7 | 96 | 300 | 1 single sample | Sternal (V.A.C.) | St. p. CABG | Discharged from hospital |
| | | | | | | |
| **Patients on micafungin** | | | | | | |
| **Patient No.** | **Body weight (kg)** | **Cum. dose (mg)** | **WS and plasma samples obtained** | **Localization and type of drainage** | **Main diagnosis** | **Clinical outcome** |
| 8 | 70 | 1,900 | Serial samples (10) | Intraabdominal (drain) | Intestinal perforation, bacterial sepsis, st. p. adhesive ileus | Deceased in ICU |

| | | | | | | |
|---|---|---|---|---|---|---|
| 9 | 50 | 600 | Serial samples (4), 1 single sample | Chest wall (drain) | St. p. LuTX, cystic fibrosis, *Candida* wound infection | Discharged from hospital |
| 10 | 90 | 300 | Serial samples (4), 1 single sample | Abdominal wall (drain) | Abdominal wall abscess, ileus, stroke, *Candida* wound infection | Discharged from hospital |
| 11 | 101 | 500 | 1 single sample | Sacral decubitus ulcer (V.A.C.) | Sacral decubitus ulcer with suspected infection (*C. lusitaniae* from swab, MIC = 0.12 mg/L) | Deceased in hospital |
| 12 | 60 | 200 | 3 single samples | Mediastinal (drain) | St. p. AVR | Deceased in hospital |
| 13 | 60 | 500 | 1 single sample | Chest wall and mediastinal (drain) | Endocarditis, st. p. MVR | Discharged from hospital |
| | | | | | | |
| | | | **Patients on caspofungin** | | | |
| **Patient No.** | **Body weight (kg)** | **Cum. dose (mg)** | **WS and plasma samples obtained** | **Localization and type of drainage** | **Main diagnosis** | **Clinical outcome** |

| | | | | | | |
|---|---|---|---|---|---|---|
| 14 | 75 | 170 | Serial samples (6) | Subphrenic - esophageal (drain) | Peritonitis, esophageal abscess, candidemia (*C. albicans* and *C. glabrata*) | Discharged from hospital |
| 15 | 100 | 490 | Serial samples (7) | Thigh (drain) | Thigh abscess with suspected infection (*C. albicans* from WS fluid, MIC = 0.004 mg/L) | Discharged from hospital |
| 16 | 85 | 370 | 1 single sample | Subcutaneous abdominal (V.A.C.) | St. p. hemihepatectomy, st. p. cholecystectomy | Deceased in ICU |
| 17 | 90 | 120 | 1 single sample | Subcutaneous abdominal (V.A.C.) | St. p. LTX | Deceased in hospital |
| 18 | 85 | 120 | 1 single sample | Abdominal wall (V.A.C.) | St. p. cholecystectomy | Discharged from hospital |
| 19 | 60 | 770 | 2 single samples | Sternal (drain) | St. p. AVR, suspected sternal wound infection (*C. blankii* from swab, MIC = 0.38 mg/L), septic shock | Deceased in ICU |

| | | | | | | |
|---|---|---|---|---|---|---|
| 20 | 90 | 70 | 2 single samples | Intraabdominal (V.A.C.) | Bacterial septic shock, peritonitis | Discharged from hospital |
| 21 | 80 | 630 | 1 single sample | Intraabdominal abscess (drain) | Necrotizing pancreatitis | Discharged from hospital |

Cum. Dose, cumulative dose; st. p., status post; WS, wound secretion; LTX, liver transplantation; TBC, tuberculosis; V.A.C., Vacuum Assisted Closure therapy; SLE, systemic lupus erythematosus; CABG, coronary artery bypass grafting; LuTX, lung transplantation; AVR, aortic valve replacement; MVR, mitral valve replacement.

From eight patients, serial WS samples and simultaneous plasma samples were obtained within a dosage interval. From seventeen patients, twenty-two solitary sample pairs were collected at different times from echinocandin infusion. Nine patients (patients 4, 5, 6, 7, 11, 16, 17, 18, and 20) underwent V.A.C. therapy.
