# Supplementary material for: Penetration of echinocandins into wound secretion of critically ill patients
Source: Infection. 2021 Apr 20;49(4):747–55. doi: 10.1007/s15010-021-01604-x (PMC8316195; doi:10.1007/s15010-021-01604-x)
Supplement: Supplementary file 2 — Supplementary file2 (PDF 129 KB) [file 15010_2021_1604_MOESM2_ESM.pdf]

**ELECTRONIC SUPPLEMENTARY MATERIAL 1**

# Penetration of Echinocandins into Wound Secretion of Critically Ill Patients

**Journal name: Infection**

Tiziana Gasperetti, René Welte, Herbert Oberacher, Jana Marx, Ingo Lorenz, Peter Schellongowski, Thomas Staudinger, Karin Burgmann, Philipp Eller, Tobias Santner, Andrea Griesmacher, Hartwig Pfisterer, Stephan Eschertzhuber, Maria Aigner, Michael Joannidis, Romuald Bellmann

Corresponding author: Romuald Bellmann, MD, associate professor,

Clinical Pharmacokinetics Unit, Division of Intensive Care and Emergency Medicine,

Department of Internal Medicine I, Medical University of Innsbruck,

Anichstrasse 35, 6020 Innsbruck, Austria, Europe,

romuald.bellmann@i-med.ac.at, Phone: 0043 512 504 81389, Fax: 0043 512 504 67 81389

**ONLINE RESOURCE 1**

**Quantification of anidulafungin (AFG) and micafungin (MFG) in wound secretion (WS) and plasma**

AFG and MFG concentrations were measured in WS and in plasma by high performance liquid chromatography and UV detection as described previously [1]. In brief, 500 µL of the samples were mixed with 500 µL of acetonitrile for AFG, with 250 µL of acetonitrile and 250 µL of 0.1% ammonium acetate buffer solution (pH 10.5) for MFG. For MFG quantification in WS, the method was slightly modified, i.e. 500 µL of WS were treated with 500 µL of acetonitrile and 500 µL of buffer solution (instead of 250 µL each) prior to the HPLC measurement. Calibrators and controls for MFG quantification were prepared by spiking WS of patients not on echinocandin treatment. As supply of blank WS was limited, ascites fluid of patients not on echinocandin treatment was used as surrogate matrix to prepare calibrators and controls for AFG quantification. The validity of this approach was proven by comparing the concentrations of quality control samples obtained with calibrations generated with both WS and ascites fluid. In all cases, deviations were within ±15%.

The process efficiencies were 50-60% for AFG and 75-85% for MFG. The lower limit of quantification (LLOQ) was 0.025 mg/L for AFG and 0.05 mg/L for MFG; bias, precision, intra-day and inter-day variability were <15%.

**Quantification of AFG and MFG in WS samples with absorption gel**

WS of patients 4, 7, and 11 was collected in V.A.C. canisters containing an absorption gel (V.A.C.®; KCI, San Antonio, USA). In this case, 0.5 g of the samples were treated in the same way as the liquid WS samples and the measured concentrations were corrected for the presence of the solid gel in the samples, by using the following formula:

$$C_{\text{ws corrected}} = (C_{\text{ws measured}}) / (\text{WS fraction})$$

Where:

$$\text{WS fraction} = (\rho_{sample} - \rho_{gel}) / (\rho_{ws} - \rho_{gel})$$

$\rho_{sample}$ is the density of the entire sample

$\rho_{gel}$ is the density of the gel

$\rho_{ws}$ is the density of WS

For calibration, the blank matrices (WS for MFG and ascites fluid for AFG) were mixed with 25 mg of gel (fixed amount, equivalent to a representative patient sample) and processed as described for the liquid samples. The process efficiencies were 40-45% for AFG and 70-80% for MFG. The LLOQ was 0.025 mg/L for AFG and 0.05 mg/L for MFG; accuracy, precision, intra-day and inter-day variability were <15%.

**Quantification of caspofungin (CAS) in WS and in plasma**

CAS was quantified by liquid chromatography-tandem mass spectrometry (LC-MS/MS). Twenty-five µL of the sample were mixed with 25 µL of internal standard solution (Caspofungin Acetate-D4 [$C_{56}H_{92}D_4N_{10}O_{19}$], Toronto Research Chemicals, North York, Canada; 1.0 mg/L). Proteins were precipitated by adding 100 µL of 0.1% formic acid in acetonitrile. After 5 minutes of ultrasonic bath and subsequent centrifugation at 1,900 × *g* for 5 minutes, the supernatant (160 µL) was mixed with 150 µL of 0.1% formic acid in acetonitrile.

The LC-MS/MS system consisted of a 1100 series HPLC pump (Agilent, Waldbronn, Germany), a CTC-PAL autosampler (CTC Analytics AG, Zwingen, Switzerland), and a QTrap 4000 mass spectrometer (Sciex, Framingham, MA, USA). Chromatographic separations were accomplished on a Zorbax SB-$C_{18}$ column (2.1 × 150 mm, 5 µm) protected by a SB-$C_{18}$ guard column (4.6 × 12.5 mm, 5 µm; both Agilent Technologies, Vienna, Austria) employing a linear gradient of 5-100% acetonitrile in aqueous 0.5% acetic acid solution within 10 minutes. The flow-rate was 300 µL/min. The injection volume was 10 µL

and the column temperature was held at 50°C. Mass spectrometry detection was carried out with electrospray ionization in positive ion mode. Multiple reaction monitoring was performed using the precursor-to-product ion transitions *m/z* 547.5 to 131.1 (quantifier) and 547.5 to 137.1 (qualifier) for CAS as well as 549.5 to 131.1 for CAS-D4.

For calibration, matrix-matched standards were used (range: 0.05-10.0 mg/L). The LLOQ was 0.1 mg/L. Validation experiments demonstrated fitness of the method. Bias and relative standard deviations were <15%. The process efficiencies were 70-80%.

**REFERENCE**

1. Welte R, Oberacher H, Schwärzler B, Joannidis M, Bellmann R. Quantification of anidulafungin and micafungin in human body fluids by high performance-liquid chromatography with UV-detection. J Chromatogr B Analyt Technol Biomed Life Sci. 2020;1139:121937. doi:10.1016/j.jchromb.2019.121937.
